# Health-related quality of life and its predictors among patients with breast cancer at Tikur Anbessa Specialized Hospital, Addis Ababa, Ethiopia

**DOI:** 10.1186/s12955-019-1239-1

**Published:** 2019-11-05

**Authors:** Selamawit Gebrehiwot Sibhat, Teferi Gedif Fenta, Beate Sander, Gebremedhin Beedemariam Gebretekle

**Affiliations:** 1 School of Pharmacy, College of Health Sciences, Addis Ababa University, Zambia Street, Addis Ababa, Ethiopia; 2 Institute of Health Policy, Management and Evaluation, University of Toronto, Toronto, Canada; 3 Toronto Health Economics and Technology Assessment (THETA) Collaborative, University Health Network, Toronto, Canada; 4 Public Health Ontario, Toronto, Ontario Canada

**Keywords:** Breast cancer, Health-related quality of life, HRQoL, EORTC-QLQ-C30, EORTC-QLQ-BR23, EQ5D, Utility

## Abstract

**Background:**

Breast cancer is the second most prevalent malignancy in Ethiopia and severely affects patients’ health-related quality of life (HRQOL). We aimed to assess HRQoL, factors influencing HRQoL, and utilities among breast cancer patients at Tikur Anbessa Specialized Hospital, Addis Ababa, Ethiopia.

**Methods:**

A hospital-based cross-sectional study was conducted in Tikur Anbessa Specialized Hospital from December 2017 to February, 2018. A total of 404 breast cancer patients were interviewed using the validated Amharic version of the European Organization for Research and Treatment of Cancer module (EORTC QLQ-C30), EORTC QLQ-BR23, and Euro Quality of Life Group’s 5-Domain Questionnaires 5 Levels (EQ-5D-5 L) instruments. Mean scores and mean differences of EORTC- QLQ-C30 and EORTC- QLQ-BR23 were calculated. One-way ANOVA test was employed to determine the significance of mean differences among dependent and independent variables while stepwise multivariate logistic regression was used to identify factors associated with the global quality of life (GQOL). Coefficients and level specific utility values obtained from a hybrid regression model for the Ethiopian population were used to compute utility values of each health state. Data was analyzed using SPSS version 23.

**Results:**

The mean age of patients was 43.94 ± 11.72 years. The mean score for GQoL and visual analog scale was 59.32 ± 22.94 and 69.94 ± 20.36, respectively while the mean utility score was 0.8 ± 0.25. Predictors of GQoL were stage of cancer (AOR = 7.94; 95% CI: 1.83–34.54), cognitive functioning (AOR = 2.38; 95% CI: 1.32–4.31), pain (AOR = 7.99; 95% CI: 4.62–13.83), financial difficulties (AOR = 2.60; 95% CI: 1.56–4.35), and future perspective (AOR = 2.08; 95% CI: 1.24–3.49).

**Conclusions:**

The overall GQoL of breast cancer patients was moderate. Targeted approaches to improve patients’ HRQoL should consider stage of cancer, cognitive functioning, pain, financial status and worries about the patient’s future health. This study also provides estimates of EQ-5D utility scores that can be used in economic evaluations.

## Introduction

Breast cancer is a growing concern worldwide as the leading cause of mortality and morbidity among women in developed and developing countries [1]. It has been predicted that the worldwide incidence of breast cancer will reach approximately 3.2 million new cases per year by 2050 [2]. Approximately 60% of deaths due to breast cancer occur in developing countries [3]. Cancer incidence in Ethiopia has been increasing over time and breast cancer is the most prevalent malignancy (30.2%) followed by cancer of the cervix (13.4%) and colorectal cancer (5.7%) among adults [4]. Consequently, cancer is emerging as a critical public health problem in Ethiopia [5, 6].

The impact of cancer, however, is far greater than the number of cases would suggest. Patients with breast cancer experience physical symptoms and psychosocial distress that adversely affect their health-related quality of life (HRQoL). The World Health Organization defined HRQoL as involving a person’s physical health, psychological state, degree of independence, social relationships, personal beliefs and environment [7]. HRQoL measures wellbeing related to or affected by the presence of a disease or treatments and it generally consists of a number of domains including physical functioning, psychological well-being (such as levels of anxiety and depression), and social support [8]. Ongoing symptoms, side effects of treatments, recurrence often result in a feeling of distress that affects physical and psychological functioning and impacts on lifestyle and social engagements of patients with breast cancer [9]. Patients receiving chemotherapy might also experience several side-effects and symptoms that negatively affect their HRQoL [10].

Deterioration of patients’ HRQoL is more pronounced in developing counties since breast cancer is diagnosed at advanced stages and as a result, treatment cannot be efficiently executed [3]. As breast cancer is the leading cause of morbidity and mortality among women with cancer in Ethiopia, HRQoL among patients with breast cancer is given minimal attention. Thus, assessing HRQoL, associated factors and utility would be helpful to inform interventions and improves patient outcome [11].

## Methods

### Study setting

The study was conducted at Tikur Anbessa Specialized Hospital (TASH), the largest teaching hospital under the administration of Addis Ababa University in Ethiopia. The hospital was established in 1972 and has more than 800 beds providing diagnostic and treatment service for about 370,000 to 400,000 patients per year. The oncology unit at TASH is the largest referral site for the country, providing service for over 60,000 patients annually. It is the sole oncology referral and radiotherapy center in the entire country [12].

### Study variables

#### **Dependent variables**: GQoL, functional scales, symptom scales

**Independent variables**:

- Socio-demographic characteristics such as age, marital status, level of education, and average monthly household income (AMHI).
- Clinical characteristics such as patient status, time since diagnosis, stage of cancer, current type of anticancer treatment and comorbid conditions.
- Functional scales
- Symptom scales

#### Study design and participants

We conducted a hospital-based cross-sectional study in patients with breast cancer at the outpatient oncology unit of TASH between December 2017 and February 2018. The sample size was calculated using the single proportion formula [13]. Due to absence of studies done using Euro Quality of Life Group’s 5-Domain Questionnaires 5 Levels (EQ-5D-5 L) and with the intention of obtaining maximum sample size, an estimate proportion of patients that have utility values above the average was considered to be 50%, was used to calculate the sample size.

$$ n=\frac{{\left(\mathrm{z}\frac{\upalpha}{2}\right)}^2\mathrm{p}\left(1-\mathrm{p}\right)}{{\mathrm{d}}^2} $$

Where: **n** = required sample size.

**Z**_**α /2**_ = 1.96 (Z = score corresponds to 95% confidence level).

**P** = proportion of patients with utility above the average.

**d**^**2**^ = margin of error (0.05)

$$ n=\frac{(1.96)^2(0.5)(0.5)}{(0.05)2}=384 $$

Considering a 5% of contingency for inappropriate and nonresponses, finally we interviewed a total of 404 patients. Since we used a face-to-face interview, all eligible patients approached were willing to participate in the study and none of the patient data were incomplete. Participants were recruited consecutively until the required sample size was reached.

Female patients pathologically diagnosed with breast cancer; both new and follow up were eligible for the study. Patients who were pregnant, critically ill (too weak to communicate, as per the assessment of oncologist), have a psychiatric disorder, can’t speak and/or read Amharic language, or are unwilling to participate in the study were excluded.

Data was collected by two trained oncology nurses working within the oncology clinic. Two days training was given for the oncology nurses focusing on; the contents of the questionnaire, the identification of patients based on the inclusion/exclusion criteria, and how to get consent. Participants were assured of anonymity and confidentiality of their information obtained in the study by excluding any personal identifier in the data collection form. They were also reassured that the report of the findings would not identify them and only the aggregate data would be reported. All the collected data were checked for completeness by the principal investigator on a daily basis.

#### Instruments and scoring

We used the validated Amharic version (official language of Ethiopia and the study area) of three data collection instruments: *EORTC QLQ-C30, EORTC QLQ-BR23 and EQ-5D-5 L* (Additional file 1)*.*

i. ***EORTC QLQ-C30:*** The EORTC QLQ-C30 consists of five functional scales (physical, emotional, role, cognitive, and social functioning), nine symptom scales (fatigue, nausea/vomiting, pain, dyspnea, insomnia, appetite loss, constipation, diarrhea and financial difficulties) and the GQoL scale, which aims to provide a multidimensional assessment of the HRQoL of patients based on 28 questions using a four-point scale. Two additional questions were used to determine the state of health on a seven-point Likert scale. Each of the multi-item scales includes a different set of items, no item occurs in more than one scale [14].
ii. ***EORTC QLQ-BR23:*** The side-effects of therapy and tumour-related symptoms in patients with breast cancer was determined and recorded using the additional EORTC QLQ-BR23 module, which consists of 23 questions distributed across eight (sexual functioning, future perspective, body image, sexual enjoyment, systemic therapy, breast symptoms, arm symptoms, and upset by hair loss) with a four-point scales; from 1 = not at all to 4 = very much [14].
iii. ***EQ-5D-5 L:***The generic EQ-5D-5 L questionnaire assessed the HRQoL across five dimensions (mobility, self-care, usual activities, pain/discomfort, and anxiety/depression), with a 5-level response (from 1 = no problem to 5 = extreme problem) and the EQ-VAS scale on which the overall state of health is marked by the patient in the form of a number (0 = worst imaginable state of health, 100 = best imaginable state of health). The utility value between the worst and best health state is on a scale from 0 to 1, where 0 denotes death and 1 denotes perfect health. EQ-5D-5 L is highly discriminatory, easy to use and can generate a single total score based on socially relevant measures of HRQoL [15].

Both EORTC QLQ-C30 and EORTC QLQ-BR23 are composed of both multi-item scales and single-item measures. Each of the multi-item scales includes a different set of items, no item occurs in more than one scale. The principle for scoring the EORTC QLQ-C30 and EORTC QLQ-BR23 scales is the same in all cases which starts with estimating the average of the items that contribute to the scale (raw score) and using a linear transformation to standardize the raw score. Scores range from 0 to 100; a higher score represents a higher (“better”) level of functioning, or a higher (“worse”) level of symptoms [14]. The two items for the scales are scored positively (i.e. “very much” is best) and therefore use the same algebraic equation as for symptom scales which is reversely coded; however, the Body Image scale uses the algebraic equation for functioning scales [16].

#### Data analysis and interpretation

Statistical analysis was undertaken using SPSS 23.0. Analyzing the data, responses were reverse coded as appropriate. Simple descriptive statistics such as frequencies, means, and standard deviations (SD) were employed to report the socio-demographic characteristics, clinical characteristics, EORTC QLQ-C30, EORTC QLQ-BR23, EQ-5D-5 L, and EQ VAS scores. Multivariable logistic regression was carried out to identify possible predicting factors for GQoL. GQoL, symptom and functional scales have been dichotomized into “Affected at any degree” and “Not affected at all”. A score below 75 (above 75 mean no problem at all) for functional and GQoL scales were defined as “Affected at any degree”. Scores above a 25 mean (below 25 indicates no symptom at all) were defined as “Affected at any degree” and binary logistic regression was conducted between the GQoL and independent variables to obtain candidate variables for multi-variable logistic regression analysis. Variables with *p*-value < 0.25 were candidate for multiple regression analysis. Due to many independent variables, forward stepwise method was used for the multivariable analysis and significance of association was determined at *p*-value < 0.05. Patient’s utility score is obtained using possible (3125) health states of patients with breast cancer defined by the 5 dimensions and disutility coefficient of general population. Thus, it was calculated using the Ethiopian general population utility value set [17]. One caveat in order is the limitation within the analysis where causality of the associations was not confirmed.

## Results

### Socio-demographic and clinical characteristics of patients

All 404 patients completed the questionnaires, i.e., there were no missing responses. Patients’ mean age was 43.94 ± 11.72 years and more than two-thirds of patients (70.2%) attended formal education. The average monthly household income (AMHI) was 2634 ± 3373 Ethiopian Birr ($1 = 27.4ETB) and one-third of patients (31.9%) had an AMHI of ≤600 ETB. The majority (89.4%) of patients were on follow-up and more than half (52.7%) were diagnosed within the past year. Regarding the severity of disease, 142 (35.1%) and 134 (33.2%) of patients with breast cancer were in cancer stage III and II, respectively. Most patients (52.5%) received surgical treatment and 318 (78.7%) had no comorbid conditions (Table 1).

**Table 1:** Socio-demographic and clinical characteristics of patients with Breast cancer at TASH, Addis Ababa, Ethiopia

Study Variables	n (%)
Age (years)	
15–24	3 (0.7)
25–54	320 (79.2)
55–64	57 (14.1)
> 65	24 (5.9)
Marital status	
Single	56 (13.9)
Married	232 (57.4)
Divorced	56 (13.9)
Widowed	60 (14.9)
Level of education	
Illiterate (neither read nor write)	92 (22.8)
Informal education	28 (6.9)
Primary education	76 (18.8)
Secondary education	123 (30.4)
Higher education (certificate, diploma, and above)	85 (21.0)
AMHI, in ETB	
≤ 600	129 (31.9)
> 600	275 (68.1)
Patient status	
New patient	43 (10.6)
Follow up	361 (89.4)
Time since diagnosis (months)	
< 12 months	213 (52.7)
13–60 months	154 (38.1)
> 61 months	37 (9.2)
Stage of cancer	
Stage I	13 (3.2)
Stage II	134 (33.2)
Stage III	142 (35.1)
Stage IV	84 (20.8)
Undefined	31 (7.7)
Current treatment	
Surgery	212 (52.5)
Chemotherapy	24 (5.9)
Hormonal therapy	139 (34.4)
Radiotherapy	29 (7.2)
Comorbid conditions	
Yes	86 (21.3)
No	318 (78.7)

#### Global quality of life

The GQoL mean score was 59.32 ± 22.94. Functional scale mean scores ranged from 67.97 ± 25.15 for physical functioning to 80.07 ± 30.08 for social functioning. All the symptom scales and items except for nausea/vomiting, dyspnea, constipation, and diarrhea scored above 25. With regard to EORTC QLQ-BR23 functioning scales/items, body image was the highest score (77.21 ± 32.09), while sexual functioning recorded the lowest score (17.78 ± 28.09). Except for breast symptoms and arm symptoms, all others scored above 25 for the symptom scales and items (Table 2).

**Table 2:** Mean score value of the EORTC QLQ-C30 and EORTC QLQ-BR23 Scales Variables of patients with Breast cancer at TASH, Addis Ababa, Ethiopia

	EORTC QLQ-C30 and EORTC QLQ-BR23 Scales	Mean ± SD
EORTC QLQ- C30	GQoL	59.32 ± 22.94
	Functional scales	
	Physical functioning	67.97 ± 25.15
	Role functioning	73.18 ± 36.19
	Emotional functioning	71.51 ± 29.74
	Cognitive functioning	78.55 ± 26.23
	Social functioning	80.07 ± 30.08
	Symptom scales and Items	
	Fatigue	42.38 ± 33.35
	Nausea and Vomiting	14.48 ± 24.96
	Pain	36.46 ± 32.91
	Dyspnoea	18.65 ± 30.69
	Insomnia	33.16 ± 39.85
	Appetite loss	36.47 ± 40.69
	Constipation	24.83 ± 35.72
	Diarrhea	4.04 ± 14.76
	Financial Difficulties	48.59 ± 44.56
EORTC QLQ-BR23	Functional scales	
	Body image	77.21 ± 32.09
	Sexual functioning	17.78 ± 28.09
	Sexual enjoyment	63.51 ± 30.98
	Future perspective	52.47 ± 43.13
	Symptom scales/items	
	Systemic therapy side effects	34.11 ± 22.59
	Breast symptoms	18.39 ± 22.71
	Arm symptoms	24.92 ± 25.06
	Upset by hair loss	26.92 ± 40.24

#### Mean differences of EORTC QLQ-C30 and EORTC QLQ-BR23 scales with socio-demographic and clinical characteristics

Family income showed a significant mean difference with GQoL, physical functioning and role functioning on the functional scales. Similarly, family income showed significant mean differences with constipation and financial difficulties on the symptom scales. The other socio-demographic variables, however, showed no significant mean difference with EORTC QLQ-C30. Moreover, patients with stage 4 cancer scored the lowest mean in GQoL, physical function and role functioning. Type of treatment showed a significant mean difference and those who were treated with radiotherapy scored the lowest mean in their GQoL, role functioning, emotional functioning and cognitive functioning. Stage 4 cancer patients had a higher mean score on fatigue, nausea and vomiting, pain, dyspnea, insomnia and appetite loss except for diarrhea and financial difficulties. Patients who took chemotherapy had a higher score in nausea and vomiting, appetite loss and diarrhea while those who took radiotherapy had a higher score on pain. However, the other symptom scales were not significant with treatment and comorbid conditions (Additional file 2). Further details are presented in Additional file 2.

#### Factors of quality of life

In multivariable analysis, five variables (stage of cancer, cognitive functioning, pain, financial difficulties, and future perspective) were found to be significantly associated with patients’ GQOL (Tables 3 and 4). Only cancer stage maintained a significant association with the socio-demographic and clinical characteristics. This implied that stage 4 breast cancer patients were 7.94 times more likely that their GQoL was affected by cancer (Table 3).

**Table 3:** Association of socio-demographic/economic factors with GQoL of patients with breast cancer at TASH, Addis Ababa, Ethiopia

	Variables	GQoL		COR (95% CI)	AOR (95% CI)
		Affected	Not affected		
Socio-demographic/economic	Educational status				
	Illiterate	69 (25.1)	23 (17.8)	1.00	
	Informal	21 (7.6)	7 (5.5)	1.00 (0.38–2.66)	
	Primary	56 (20.4)	20 (15.5)	0.93 (0.47–1.87)	
	Secondary	84 (30.5)	39 (30.2)	0.72 (0.39–1.32)	
	Higher	45 (16.4)	40 (31.0)	0.38 (0.19–0.71)	
	AMHI				
	≤600	100 (36.4)	29 (22.5)	1.00	
	> 600	175 (63.6)	100 (77.5)	0.51 (0.31–0.82)	
Clinical characteristics	Stage of cancer				
	Stage 1	7 (2.6)	6 (4.7)	1.00	
	Stage 2	86 (31.3)	48 (37.3)	1.54 (0.49–4.83)	3.09 (0.79–12.09)
	Stage 3	93 (33.8)	49 (38.0)	1.63 (0.52–5.11)	3.08 (0.79–12.01)
	Stage 4	71 (25.8)	13 (10.0)	4.68 (1.35–16.18)	7.94 (1.83–34.54) *
	Undefined	18 (6.5)	13 (10.0)	1.19 (0.32–4.37)	2.04 (0.43–9.61)
	Current Treatment				
	Chemo therapy	151 (54.9)	61 (47.3)	1.00	
	Surgery	15 (5.5)	9 (6.9)	0.67 (0.28–1.62)	
	Hormonal therapy	85 (30.9)	54 (41.9)	0.64 (0.40–0.99)	
	Radiotherapy	24 (8.7)	5 (3.9)	1.94 (0.71–5.32)

**Table 4:** Association between (EORTC QLQ-C30, EORTC QLQ-BR23) functioning and symptom scales with GQoL of patients with breast cancer at TASH, Addis Ababa, Ethiopia

	Variable		GQOL		COR (95% CI)	AOR (95% CI)
			Affected	Not affected		
EORTC QLQ C-30	Functional scales					
	Physical functioning	Affected	187 (68.0)	46 (35.7)	3.83 (2.47–5.96)	
		Not affected	88 (32.0)	83 (64.3)	1.00	
	Role Functioning	Affected	139 (50.5)	18 (14.0)	6.30 (3.63–10.94)	
		Not affected	136 (49.5)	111 (86.0)	1.00	
	Emotional Functioning	Affected	134 (48.7)	30 (23.3)	3.14 (1.96–5.03)	
		Not affected	141 (51.3)	99 (76.7)	1.00	
	Cognitive Functioning	Affected	121 (44.0)	23 (17.8)	3.62 (2.18–6.03)	2.38 (1.32–4.31)*
		Not affected	154 (56.0)	106 (82.2)	1.00	1.00
	Social Functioning	Affected	104 (37.8)	27 (20.9)	2.29 (1.41–3.75)	
		Not affected	171 (62.2)	102 (79.1)	1.00	
	Symptom scales					
	Fatigue	Affected	204 (74.2)	45 (34.9)	5.36 (3.41–8.43)	
		Not affected	71 (25.8)	84 (65.1)	1.00	
	Nausea and Vomiting	Affected	87 (31.6)	14 (10.9)	3.80 (2.07–6.99)	
		Not affected	188 (68.4)	115 (89.1)	1.00	
	Pain	Affected	195 (70.9)	26 (20.2)	9.66 (5.84–15.96)	7.99 (4.62–13.83)*
		Not affected	80 (29.1)	103 (79.8)	1.00	1.00
	Dyspnoea	Affected	113 (41.1)	18 (14.0)	4.30 (2.47–7.48)	
		Not affected	162 (58.9)	111 (86.0)	1.00	
	Insomnia	Affected	152 (55.3)	37 (28.7)	3.07 (1.96–4.82)	
		Not affected	123 (44.7)	92 (71.3)	1.00	
	Appetite loss	Affected	166 (60.4)	38 (29.5)	3.65 (2.33–5.72)	
		Not affected	109 (39.6)	91 (70.5)	1.00	
	Constipation	Affected	122 (44.4)	35 (27.1)	2.14 (1.36–3.38)	
		Not affected	153 (55.6)	94 (72.9)	1.00	
	Diarrhea	Affected	27 (9.8)	7 (5.4)	1.89 (0.80–4.48)	
		Not affected	248 (90.2)	122 (94.6)	1.00	
	Financial Difficulties	Affected	187 (68.0)	54 (41.9)	2.95 (1.92–4.55)	2.60 (1.56–4.35)*
		Not affected	88 (32.0)	75 (58.1)	1.00	1.00
EORTC QLQ BR-23	Functional scales					
	Sexual functioning	Affected	70 (25.5)	50 (38.8)	0.54 (0.35–0.84)	
		Not affected	205 (74.5)	79 (61.2)	1.00	
	Future Perspective	Affected	181 (65.8)	61 (47.3)	2.15 (1.40–3.29)	2.08 (1.24–3.49)*
		Not affected	94 (34.2)	68 (52.7)	1.00	1.00
	Symptom scales					
	Systemic therapy side effects	Affected	184 (66.9)	44 (34.1)	3.91 (2.51–6.08)	
		Not affected	91 (33.1)	85 (65.9)	1.00	
	Breast Symptoms	Affected	109 (39.6)	24 (18.6)	2.87 (1.73–4.76)	
		Not affected	166 (60.4)	105 (81.4)	1.00	
	Arm symptoms	Affected	119 (43.3)	28 (21.7)	2.75 (1.70–4.46)	
		Not affected	156 (56.7)	101 (78.3)	1.00	

AOR Adjusted Odd’s Ratio, COR crudes Odd’s Ratio

For EORTC QLQ-C30, only cognitive functioning was significant. Thus, patients GQoL was 2.38 times more likely to be affected if they reported problems with cognitive functioning. Among the symptom scale variables, pain and financial difficulties maintained their association in the multivariable analysis. Patients GQoL was 8 times more likely to be affected if they reported problems with pain. Patients GQoL was 2.60 times more likely to be affected if they reported problems with financial difficulties. For the breast specific EORTC QLQ-BR23 of the functional scales, only future perspective maintained the association in the multi-variable analysis (AOR = 2.08;95% CI: 1.24–3.49) (Table 4).

#### EQ-5D dimensions affected by breast Cancer and utility score

**Fig. 1:**
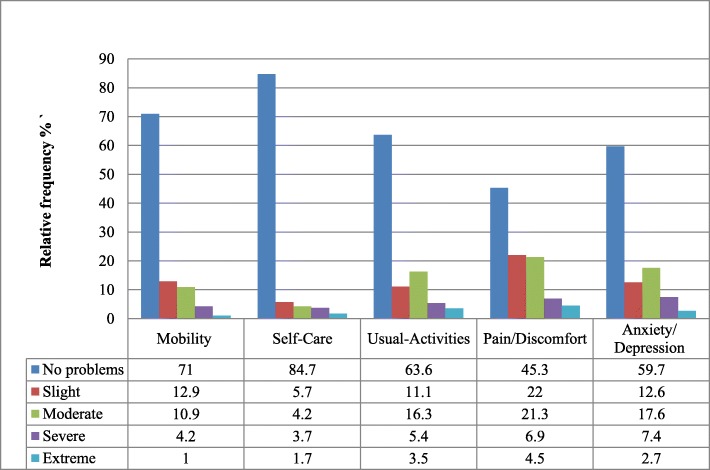
Frequency distribution of the five-dimensional EQ-5D-5 L questionnaire of patients with Breast cancer at TASH, Addis Ababa, Ethiopia, 2018

For the EQ-5D-5 L, except for pain variable, more than half of the patients had no problem in any of the five dimensions. The study showed that 23.8, 4.2, and 1% of the patients reported slight to moderate, severe mobility problem, and unable to walk, respectively. According to the study, 9.9% of them reported a slight to moderate self-care problem while 1.7% of them were unable to wash or dress themselves. Regarding daily activities, 27.4% of the patients reported that they experienced slight to moderate problems in their daily activity with 3.5% were unable to do their usual activities. 43.3% of the patients reported that they suffered slight to moderate pain, 6.9% suffered a severe pain and 4.5% suffered an extreme pain. Considering depression/anxiety, 30.2, 7.4, and 2.7% of the patients suffered a slight to moderate, severe, and an extreme anxiety/depression, respectively (Fig. 1).The mean score for the EQ-VAS was 69.94 ± 20.36, while the mean utility score was found to be 0.8 ± 0.25., which translates to patients with breast cancer preferred to trade-off 2 years of live and preferred to live 8 years in full health compared to living 10 years with their current health status.

## Discussion

The purpose of the study was to assess HRQoL, predicting factors and utility among patients with breast cancer in TASH. In the assessment of functioning scales, the lowest score was found for physical and sexual functioning. Highest symptom scales were found for fatigue, pain, loss of appetite and systemic therapy side effects were reported. The mean score for GQoL was 59.32 ± 22.94 which is almost similar to studies conducted in Iran, Central rural India, Germany and Lebanon [10, 18–20]. Our finding, however, was lower than the EORTC reference value (61.8 ± 24.6) [21] and studies conducted in South India, Australia, UK, Bahrain, Jordan, and Latin America [21–26]. This could be due to limited understanding of the disease, the lengthy process of referral to the country’s only specialized center, and late presentation; with most patients at the center diagnosed in a metastasized level which makes the disease incurable [27].

Physical and cognitive functioning were lower than the reference value, whereas role, emotional and social functioning were higher than the reference value [28]. Symptom scales of the EORTC-C30 were higher than the reference value except for diarrhea, which implied that the patient with breast cancer were very symptomatic. Fatigue and financial difficulties were the highest complaints. The mean score of financial difficulties of this study were higher than studies conducted in Nepal, Iran, Kuwait and Nigeria [10, 29–31]. The current study also showed that household income had a significant mean difference with GQoL, and 31.9% of the study participants were below the poverty line [32]. And since TASH is a destination for patients from every corner of the country, transport and hospitality fees in Addis Ababa are not easily affordable, and this could have contributed to the higher scores of financial difficulties [6].

Regarding the breast specific assessment tool, the mean results of the functional and symptom subscales in this study were higher than results of the studies conducted in Kuwait and Morocco [30, 33] but lower than studies conducted in south India, Germany, UK, Bahrain, Iran, and Latin [10, 19, 22, 24–26]. The burden of breast cancer in Ethiopian women may be higher because of limited healthcare access with only a single radiotherapy center in the country [6]. This might exacerbate symptoms because of long waiting times. This might be also attributed to a limited psychological support for breast cancer patients in the Ethiopian health care system and community.

Pain was the major predictor factor of GQoL; the significant mean difference showed that stage 4 breast cancer patients and patients who were on chemotherapy and radiotherapy had higher mean result. The current results from the EQ-5D-5 L also support that pain is a major complaint among breast cancer patients in TASH. A study conducted in Ethiopia also reported the inadequacy of cancer pain management [34]. Another study also suggested that early symptom screening should be incorporated into nursing assessment procedures for a better outcome [35].

Cancer Stage 4 was found to be one of the GQoL predictors. A significant mean difference was also seen between GQoL and stage 4 patients. The association between cancer stage and GQoL were similar to a study conducted in Bahrain [25]. Considering the access of cancer treatment in Ethiopia, Which is accompanied by long waiting time, it is difficult for a great majority of the population to access cancer treatment services. In Addition to that, the low awareness of cancer signs and symptoms, inadequate screening and early detection and treatment services, inadequate diagnostic facilities and country’s very few cancer specialists, also results in many potentially curable tumors to progress to incurable stages [6].

The present study indicated that cognitive functioning was one of the GQoL predictors and showed significant mean difference between cognitive functioning and treatment, which mirrors a study conducted in Tunisia [36]. Cognitive functioning of patients could be compromised due to chemotherapy, pain and disease burden of patients [37]. Patients at TASH could benefit from a follow up of investigation of cognitive functioning.

Patients whose physical condition or medical treatment caused them financial difficulties were a GQoL predictor. A study conducted in Kuwait also showed the importance of financial difficulties [30]. Future perspective was found to be another predictor of the GQoL. This finding was in contrary with the study done in Kuwait, where about two-thirds of the patients were optimistic about their future health [30]. This difference of future perspective could attribute to the lower awareness, improper understanding of the disease, associated stigma and sense of hopelessness of Ethiopian cancer patients [27].

The health state determined using EQ-VAS was found to be higher than a study conducted in Germany and lower than a study conducted in Zimbabwe [19, 38]. And the utility mean score value of the patients with breast cancer was estimated to be 0.8, which is almost similar with Finnish populations (0.89) [39]. Thus, the utility values have been used to make health economic evaluations and decisions relevant for better health outcome of patients [40]. The current research can be used to inform patient care and future economic evaluation for breast cancer patients.

Therefore, the present study will fill the knowledge gap about the impact of socio-demographic and clinical factors on HRQoL among patients with breast cancer in the study setting. The study used a large sample size and validated measurement tools to assess the HRQoL. Furthermore, it will help healthcare providers to recognize the causes that affect HRQoL and identify the aspects of patient treatment protocol that needs to be enhanced to improve their HRQoL since its assessment is used to measure the outcome of medical intervention. It will mainly help for economic evaluation of existing and new chemotherapy drugs for patients with breast cancer.

Despite all its strengths, the study has certain limitations. Since the study was a cross-sectional study, it might limit assessment of prognosis of the patients. In addition, the study was conducted in a single setting, which might be difficult to make a generalization for the country.

## Conclusion

The GQoL of breast cancer patients was below the population reference but comparable to other studies. The utility mean score was estimated to be above average (u = 0.8). HRQoL could be used to continuously monitor outcomes and focus should be given to pain management, and strengthening the insurance agency to improve access and affordability.

## Additional files

**Additional file 1.** Validated Amharic version of three data collection instruments.

**Additional file 2. Table S1.** Mean differences of EORTC QLQ-C30 functional scale with Socio-demographic/socio-economic characteristics of patients with Breast cancer at TASH, Addis Ababa, Ethiopia, 2018. **Table S2.** Mean differences of EORTC QLQ-C30 functional scale with clinical characteristics of patients with Breast cancer at TASH, Addis Ababa, Ethiopia, 2018. **Table S3.** Mean differences of EORTC QLQ-C30 symptom scale with socio-demographic/socio-economic characteristics of patients with breast cancer at TASH, Addis Ababa, Ethiopia, 2018. **Table S4.** Mean differences of EORTC QLQ-C30 symptom scale with clinical characteristics of patients with breast cancer at TASH, Addis Ababa, Ethiopia, 2018. **Table S5.** Mean differences of EORTC QLQ-BR23 functional scale with socio-demographic/socio-economic characteristics of patients with breast cancer at TASH, Addis Ababa, Ethiopia, 2018. **Table S6.** Mean differences in EORTC QLQ-BR23 functional scale with clinical characteristics of patients with breast cancer at TASH, Addis Ababa, Ethiopia, 2018. **Table S7.** Mean differences in EORTC QLQ-BR23 symptom scale with clinical characteristics of patients with breast cancer at TASH, Addis Ababa, Ethiopia, 2018. **Table S8.** Mean differences of EORTC QLQ-BR23 symptom scale with socio-demographic characteristics/socio-economic of patients with breast cancer at TASH, Addis Ababa, Ethiopia, 2018.

## Data Availability

For data protection the data set is not publicly accessible. However, data can be accessed from the primary or corresponding author upon reasonable request with a signature of data privacy form.

## Abbreviations

AMHI: Average Monthly Household Income
EORTC QLQ-BR23: European Organization for Research and Treatment of Cancer-Breast Module
EORTC QLQ-C30: European Organization for Research and Treatment of Cancer
EQ5D-5 L: Euro Quality of Life Group’s 5-Domain Questionnaires 5 Levels
EQ-VAS: Euro Quality of Life Group’s Visual Analog Scale
ETB: Ethiopian Birr
GQoL: Global Quality of Life
HRQoL: Health-related Quality of Life
TASH: Tikur Anbessa Specialized Hospital
UK: United Kingdom
