# Supplementary material for: Health-related quality of life and its predictors among patients with breast cancer at Tikur Anbessa Specialized Hospital, Addis Ababa, Ethiopia
Source: Health Qual Life Outcomes. 2019 Nov 5;17:165. doi: 10.1186/s12955-019-1239-1 (PMC6833153; doi:10.1186/s12955-019-1239-1)
Supplement: Supplementary file 1 — Additional file 1. Validated Amharic version of three data collection instruments. [file 12955_2019_1239_MOESM1_ESM.pdf]

Addis Ababa University

College of Health Sciences

School of Pharmacy

Department of Pharmaceutical and Social Pharmacy

Questionnaire for data collection on Assessment of health related quality of life and its associated factors among women breast cancer patients at Tikur Anbessa Specialized Referral Hospital, Addis Ababa, Ethiopia

Verbal consent form before conducting interview

Hello, my name is ______________________________. I am working in college of health sciences, School of Pharmacy, Addis Ababa University and this study is for fulfillment masters in pharmaco-epidemiology and social pharmacy. I would like to ask you a few questions regarding your health status and services provided to you. The interview would take a maximum of 15 minutes of your time. The purpose of this study is to assess the quality of life of patients with breast cancer at Tikur Anbassa Specialized Hospital, Addis Ababa, Ethiopia. This will be helpful in improving the service delivery the choice of treatment and give priority to the unmet needs. Your participation is completely voluntary. You can refuse to answer any questions and/or withdraw from the study at any time without a problem to you or the services you get in the hospital. All your responses will remain strictly confidential: the hospital staff will not have access to your responses, your name will not appear on the interview guide (will not be recorded), and your responses will not be linked to your identity at any time.

Are you volunteer to participate in the study?

Yes ☐ No ☐

If Yes, Continue to the Next Page

If No, Skip to the next Respondent

Section 1:

Socio-demographic characteristics	Response
1.1 Age	______________years
1.2 Marital Status	single □ Divorced □ Married □ Widowed □
1.3 Level of education	Unable to read and write□<br>Able to read and write □<br>Primary school □<br>Secondary school and preparatory school □<br>Higher education (first degree and above) □
1.4 Employment status	Government employee □<br>Employee of private company □<br>Merchant □ Retired □<br>Farmer□ Student □ House wife □<br>Other, please specify<br>______________________________
1.5 Monthly average family income in birr	________________

Section 2: Medical Characteristics (to be filled through chart review by data collectors)

2.1 current Cancer Stage	Stage I □ Stage II □<br>Stage III □ Stage IV □<br>If Not mentioned, please write the card number __________________________
2.2 Disease status	No Evidence of status □ Local □<br>Metastatic □
2.3 Current type of anticancer treatment (within the period of the data collection) (more than one answer possible)	Surgery □ chemotherapy only □<br>radiation only □
2.4 Please specify known comorbid condition	________________________

EORTC QLQ-C30 (version 3)

We are interested in some things about you and your health. Please answer all of the questions yourself by circling the number that best applies to you. There are no "right" or "wrong" answers. The information that you provide will remain strictly confidential.

Please fill in your initials:

Your birthdate (Day, Month, Year):

Today's date (Day, Month, Year): 31

		Not at All	A Little	Quite a Bit	Very Much
1.	Do you have any trouble doing strenuous activities, like carrying a heavy shopping bag or a suitcase?	1	2	3	4
2.	Do you have any trouble taking a long walk?	1	2	3	4
3.	Do you have any trouble taking a short walk outside of the house?	1	2	3	4
4.	Do you need to stay in bed or a chair during the day?	1	2	3	4
5.	Do you need help with eating, dressing, washing yourself or using the toilet?	1	2	3	4

During the past week:

		Not at All	A Little	Quite a Bit	Very Much
6.	Were you limited in doing either your work or other daily activities?	1	2	3	4
7.	Were you limited in pursuing your hobbies or other leisure time activities?	1	2	3	4
8.	Were you short of breath?	1	2	3	4
9.	Have you had pain?	1	2	3	4
10.	Did you need to rest?	1	2	3	4
11.	Have you had trouble sleeping?	1	2	3	4
12.	Have you felt weak?	1	2	3	4
13.	Have you lacked appetite?	1	2	3	4
14.	Have you felt nauseated?	1	2	3	4
15.	Have you vomited?	1	2	3	4
16.	Have you been constipated?	1	2	3	4

Please go on to the next page

**During the past week:**	**Not at All**	**A Little**	**Quite a Bit**	**Very Much**
17. Have you had diarrhea?	1	2	3	4
18. Were you tired?	1	2	3	4
19. Did pain interfere with your daily activities?	1	2	3	4
20. Have you had difficulty in concentrating on things, like reading a newspaper or watching television?	1	2	3	4
21. Did you feel tense?	1	2	3	4
22. Did you worry?	1	2	3	4
23. Did you feel irritable?	1	2	3	4
24. Did you feel depressed?	1	2	3	4
25. Have you had difficulty remembering things?	1	2	3	4
26. Has your physical condition or medical treatment interfered with your family life?	1	2	3	4
27. Has your physical condition or medical treatment interfered with your social activities?	1	2	3	4
28. Has your physical condition or medical treatment caused you financial difficulties?	1	2	3	4

For the following questions please circle the number between 1 and 7 that best applies to you

29. How would you rate your overall health during the past week?

1	2	3	4	5	6	7
Very poor						Excellent

30. How would you rate your overall quality of life during the past week?

1	2	3	4	5	6	7
Very poor						Excellent

© Copyright 1995 EORTC Quality of Life Group. All rights reserved. Version 3.0

EORTC QLQ - BR23

Patients sometimes report that they have the following symptoms or problems. Please indicate the extent to which you have experienced these symptoms or problems during the past week.

**During the past week:**	**Not at All**	**A Little**	**Quite a Bit**	**Very Much**
31. Did you have a dry mouth?	1	2	3	4
32. Did food and drink taste different than usual?	1	2	3	4
33. Were your eyes painful, irritated or watery?	1	2	3	4
34. Have you lost any hair?	1	2	3	4
35. Answer this question only if you had any hair loss: Were you upset by the loss of your hair?	1	2	3	4
36. Did you feel ill or unwell?	1	2	3	4
37. Did you have hot flushes?	1	2	3	4
38. Did you have headaches?	1	2	3	4
39. Have you felt physically less attractive as a result of your disease or treatment?	1	2	3	4
40. Have you been feeling less feminine as a result of your disease or treatment?	1	2	3	4
41. Did you find it difficult to look at yourself naked?	1	2	3	4
42. Have you been dissatisfied with your body?	1	2	3	4
43. Were you worried about your health in the future?	1	2	3	4

**During the past <u>four</u> weeks:**	**Not at All**	**A Little**	**Quite a Bit**	**Very Much**
44. To what extent were you interested in sex?	1	2	3	4
45. To what extent were you sexually active? (with or without intercourse)	1	2	3	4
46. Answer this question only if you have been sexually active: To what extent was sex enjoyable for you?	1	2	3	4

Please go on to the next page

SPECIMEN

**During the past week:**	**Not at All**	**A Little**	**Quite a Bit**	**Very Much**
47. Did you have any pain in your arm or shoulder?	1	2	3	4
48. Did you have a swollen arm or hand?	1	2	3	4
49. Was it difficult to raise your arm or to move it sideways?	1	2	3	4
50. Have you had any pain in the area of your affected breast?	1	2	3	4
51. Was the area of your affected breast swollen?	1	2	3	4
52. Was the area of your affected breast oversensitive?	1	2	3	4
53. Have you had skin problems on or in the area of your affected breast (e.g., itchy, dry, flaky)?	1	2	3	4

SPECIMEN

© Copyright 1994 EORTC Quality of Life Group. All rights reserved. Version 1.0

Health Questionnaire

English version for the UK

UK (English) © 2009 EuroQol Group EQ-5D™ is a trade mark of the EuroQol Group

Under each heading, please tick the ONE box that best describes your health TODAY.

MOBILITY

I have no problems in walking about ❑

I have slight problems in walking about ❑

I have moderate problems in walking about ❑

I have severe problems in walking about ❑

I am unable to walk about ❑

SELF-CARE

I have no problems washing or dressing myself ❑

I have slight problems washing or dressing myself ❑

I have moderate problems washing or dressing myself ❑

I have severe problems washing or dressing myself ❑

I am unable to wash or dress myself ❑

USUAL ACTIVITIES *(e.g. work, study, housework, family or leisure activities)*

I have no problems doing my usual activities ❑

I have slight problems doing my usual activities ❑

I have moderate problems doing my usual activities ❑

I have severe problems doing my usual activities ❑

I am unable to do my usual activities ❑

PAIN / DISCOMFORT

I have no pain or discomfort ❑

I have slight pain or discomfort ❑

I have moderate pain or discomfort ❑

I have severe pain or discomfort ❑

I have extreme pain or discomfort ❑

ANXIETY / DEPRESSION

I am not anxious or depressed ❑

I am slightly anxious or depressed ❑

I am moderately anxious or depressed ❑

I am severely anxious or depressed ❑

I am extremely anxious or depressed ❑

UK (English) © 2009 EuroQol Group EQ-5D™ is a trade mark of the EuroQol Group

- We would like to know how good or bad your health is TODAY.

- This scale is numbered from 0 to 100.

- 100 means the <u>best</u> health you can imagine.
  0 means the <u>worst</u> health you can imagine.

- Mark an X on the scale to indicate how your health is TODAY.

- Now, please write the number you marked on the scale in the box below.

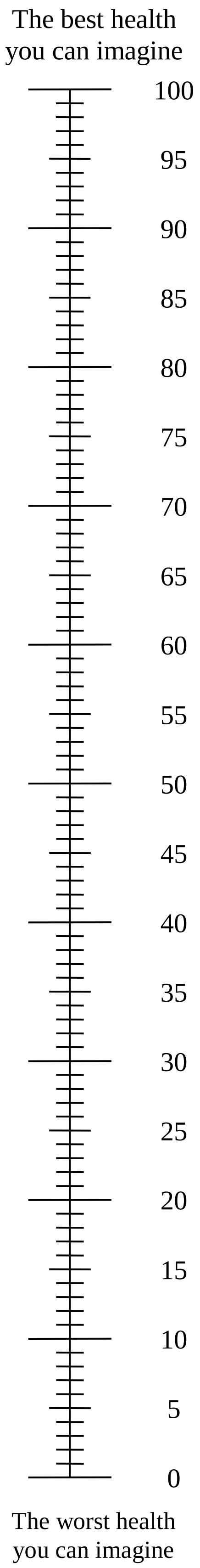

YOUR HEALTH TODAY = [ ]

UK (English) © 2009 EuroQol Group EQ-5D™ is a trade mark of the EuroQol Group
