# Supplementary material for: Health-related quality of life and its predictors among patients with breast cancer at Tikur Anbessa Specialized Hospital, Addis Ababa, Ethiopia
Source: Health Qual Life Outcomes. 2019 Nov 5;17:165. doi: 10.1186/s12955-019-1239-1 (PMC6833153; doi:10.1186/s12955-019-1239-1)
Supplement: Supplementary file 2 — Additional file 2. Table S1. Mean differences of EORTC QLQ-C30 functional scale with Socio-demographic/socio-economic characteristics of patients with Breast cancer at TASH, Addis Ababa, Ethiopia, 2018. Table S2. Mean differences of EORTC QLQ-C30 functional scale with clinical characteristics of patients with Breast cancer at TASH, Addis Ababa, Ethiopia, 2018. Table S3. Mean differences of EORTC QLQ-C30 symptom scale with socio-demographic/socio-economic characteristics of patients with breast cancer at TASH, Addis Ababa, Ethiopia, 2018. Table S4. Mean differences of EORTC QLQ-C30 symptom scale with clinical characteristics of patients with breast cancer at TASH, Addis Ababa, Ethiopia, 2018. Table S5. Mean differences of EORTC QLQ-BR23 functional scale with socio-demographic/socio-economic characteristics of patients with breast cancer at TASH, Addis Ababa, Ethiopia, 2018. Table S6. Mean differences in EORTC QLQ-BR23 functional scale with clinical characteristics of patients with breast cancer at TASH, Addis Ababa, Ethiopia, 2018. Table S7. Mean differences in EORTC QLQ-BR23 symptom scale with clinical characteristics of patients with breast cancer at TASH, Addis Ababa, Ethiopia, 2018. Table S8. Mean differences of EORTC QLQ-BR23 symptom scale with socio-demographic characteristics/socio-economic of patients with breast cancer at TASH, Addis Ababa, Ethiopia, 2018. [file 12955_2019_1239_MOESM2_ESM.docx]

Table S1: Mean differences of EORTC QLQ-C30 functional scale with Socio-demographic/socio-economic characteristics of patients with Breast cancer at TASH, Addis Ababa, Ethiopia, 2018.

|  | | | | | GQoL | | | PF | | | RF | | | EF | | | CF | | | SF | | |  |  |
| --- | --- | --- | --- | --- | --- | --- | --- | --- | --- | --- | --- | --- | --- | --- | --- | --- | --- | --- | --- | --- | --- | --- | --- | --- |
| Age(years) | | | | | | | | | | | | | | | | | | | | | | |  |  |
|  | | 15-24 | | | 52.8 ± 4.8 | | | 88.9 ± 10.2 | | | 94.4 ± 9.6 | | | 52.8 ± 19.3 | | | 88.9 ± 9.6 | | | 61.1 ± 34.7 | | |  |  |
|  | | 25-54 | | | 59.5 ± 23.6 | | | 69.7 ± 24.4 | | | 73.2 ± 35.8 | | | 71.9 ± 29.2 | | | 79.2 ± 26.1 | | | 80.5 ± 30.3 | | |  |  |
|  | | 55-64 | | | 59.3 ± 21.0 | | | 64.6 ± 25.7 | | | 74.8 ± 36.6 | | | 71.2 ± 32.3 | | | 74.3 ± 27.6 | | | 79.2 ± 28.7 | | |  |  |
|  | | >65 | | | 57.3 ± 19.5 | | | 50.5 ± 27.8 | | | 65.9 ± 41.5 | | | 69.8 ± 32.4 | | | 79.2 ± 26.1 | | | 79.1 ± 30.4 | | |  |  |
|  | | | *p*-value |  | | 0.928 | | | 0.001* | | | 0.551 | | | 0.725 | | | 0.539 | | | 0.728 | | |  |
| Marital status | | | | | | | | | | | | | | | | | | | | | | |  |  |
|  | | Single | | | 58.5 ± 24.6 | | | 69.3 ± 23.7 | | | 75.3 ± 36.1 | | | 75.7 ± 27.6 | | | 86.0 ± 19.5 | | | 72.6 ± 36.6 | | |  |  |
|  | | Married | | | 60.2 ± 22.6 | | | 68.6 ± 24.7 | | | 72.6 ± 35.6 | | | 70.4 ± 30.6 | | | 76.7 ± 27.9 | | | 80.8 ± 28.4 | | |  |  |
|  | | Divorced | | | 60.6 ± 24.2 | | | 72.1 ± 23.2 | | | 75.3 ± 34.8 | | | 73.5 ± 26.4 | | | 80.3 ± 22.3 | | | 81.8 ± 30.2 | | |  |  |
|  | | Widowed | | | 55.7 ± 21.8 | | | 60.2 ± 28.6 | | | 71.4 ± 40.1 | | | 70.1 ± 31.5 | | | 76.9 ± 27.3 | | | 82.5 ± 29.5 | | |  |  |
|  | | | *p*-value | |  | | 0.563 | | | 0.055 | | | 0.901 | | | 0.604 | | | 0.104 | | | 0.245 | | |
| Level of education | | | | | | | | | | | | | | | | | | | | | | |  |  |
|  | | Illiterate | | | 53.3 ± 23.5 | | | 62.0 ± 26.2 | | | 64.5 ± 36.4 | | | 66.2 ± 33.1 | | | 73.2 ± 29.3 | | | 71.9 ± 32.2 | | |  |  |
|  | | Informal  Education | | | 53.9 ± 19.4 | | | 65.7 ± 23.7 | | | 77.9 ± 31.4 | | | 76.8 ± 26.9 | | | 83.9 ± 20.0 | | | 89.9 ± 21.4 | | |  |  |
|  | | Primary  education | | | 58.0 ± 21.6 | | | 64.6 ± 27.9 | | | 73.5 ± 37.6 | | | 70.7 ± 27.4 | | | 78.1 ± 24.6 | | | 76.3 ± 33.9 | | |  |  |
|  | | Secondary  Education | | | 59.5 ± 23.5 | | | 69.6 ± 26.4 | | | 73.6 ± 37.5 | | | 72.7 ±28.7 | | | 81.0 ± 24.8 | | | 83.9 ± 27.5 | | |  |  |
|  | | Higher  education | | | 68.5 ± 21.3 | | | 75.8 ± 16.8 | | | 80.2 ± 32.9 | | | 74.5 ± 30.1 | | | 79.4 ± 27.4 | | | 83.5 ± 28.2 | | |  |  |
|  | | | *p*-value | | 0.000* | | | 0.004* | | | 0.060 | | | 0.297 | | | 0.182 | | | 0.007* | | |  |  |
| AMHI, in ETB | | | | | | | | | | | | | | | | | | | | | | |  |  |
|  | ≤600 | | | | 50.6 ± 28.4 | | | 63.9 ± 26.5 | | | 66.7 ± 38.5 | | | 69.9 ± 29.4 | | | 77.8 ±24.0 | | | 76.9 ±31.5 | | |  |  |
|  | >600 | | | | 63.4 ± 21.4 | | | 69.9 ± 24.3 | | | 76.2 ± 34.7 | | | 72.2 ± 29.9 | | | 78.9 ±27.2 | | | 81.6 ±29.3 | | |  |  |
|  | | | *p*-value | | 0.000* | | | 0.027* | | | 0.013* | | | 0.473 | | | 0.687 | | | 0.143 | | |  |  |

PF=Physical Functioning, RF=Role functioning, EF=Emotional functioning, CF =Cognitive functioning, SF=Social functioning

*The mean difference is significant< 0.05

Table S2: Mean differences of EORTC QLQ-C30 functional scale with clinical characteristics of patients with Breast cancer at TASH, Addis Ababa, Ethiopia, 2018.

|  | | | GQoL | | | PH | | | | RF | | | EF | | | CF | | | SF | | |  |  |
| --- | --- | --- | --- | --- | --- | --- | --- | --- | --- | --- | --- | --- | --- | --- | --- | --- | --- | --- | --- | --- | --- | --- | --- |
| Patient status | | | | | | | | | | | | | | | | | | | | | |  |  |
|  | New patient | | 59.1 ± 22.7 | | | 69.4 ± 29.4 | | | | 65.9 ± 40.3 | | | 59.7 ± 32.5 | | | 80.6 ± 24.1 | | | 84.1 ± 26.5 | | |  |  |
|  | Follow up | | 59.3 ± 23.0 | | | 67.8 ± 24.6 | | | | 74.0 ± 35.6 | | | 72.9 ± 29.1 | | | 78.3 ± 26.5 | | | 79.6 ± 30.5 | | |  |  |
|  | | *p*-value |  | 0.948 | | | | 0.682 | | | 0.162 | | | 0.006* | | | 0.584 | | | 0.353 | | |  |
| Time since diagnosis (months) | | | | | | | | | | | | | | | | | | | | | |  |  |
|  | <12 | | 58.8 ± 22.9 | | | 67.9 ± 24.7 | | | | 69.1 ± 37.5 | | | 69.1 ± 29.9 | | | 78.1 ± 24.7 | | | 79.9 ± 30.3 | | |  |  |
|  | 13-60 | | 59.6 ± 23.3 | | | 68.1 ± 26.5 | | | | 77.9 ± 34.1 | | | 74.0 ± 30.1 | | | 78.2 ± 28.8 | | | 81.1 ± 28.8 | | |  |  |
|  | >61 | | 61.0 ± 21.9 | | | 67.2 ± 22.2 | | | | 77.0 ± 34.9 | | | 75.2 ± 26.9 | | | 81.9 ± 23.3 | | | 77.0 ± 34.8 | | |  |  |
|  | | *p*-value | 0.843 | | | 0.980 | | | | 0.055 | | | 0.209 | | | 0.706 | | | 0.759 | | |  |  |
| Stage of cancer | | | | | | | | | | | | | | | | | | | | | |  |  |
|  | Stage 1 | | 62.2± 25.6 | | | | 68.7 ± 22.8 | | | 82.0 ± 24.9 | | | 62.2 ± 31.8 | | | 85.9 ± 22.4 | | | 76.9 ± 25.9 | | |  |  |
|  | Stage 2 | | 64.4 ±19.1 | | | | 73.4 ± 20.8 | | | 81.1 ± 31.1 | | | 74.3 ± 28.8 | | | 77.9 ±26. 4 | | | 84.2 ± 26.2 | | |  |  |
|  | Stage 3 | | 61.5 ± 22.5 | | | | 69.5 ± 23.9 | | | 73.6 ± 35.2 | | | 72.9 ± 28.4 | | | 78.6 ± 26.9 | | | 79.8 ± 30.9 | | |  |  |
|  | Stage 4 | | 46.0 ± 23.9 | | | | 54.9 ± 31.3 | | | 54.8 ± 42.8 | | | 64.9 ± 32.4 | | | 76.2 ± 27.2 | | | 73.8 ± 33.5 | | |  |  |
|  | Unknown | | 62.1 ± 24.1 | | | | 72.5 ± 17.6 | | | 83.3 ± 27.9 | | | 74.5 ± 30.0 | | | 83.9 ± 20.8 | | | 81.7 ± 32.3 | | |  |  |
|  | | *p*-value | 0.000* | | | | 0.000* | | | 0.000* | | | 0.127 | | | 0.553 | | | 0.170 | | |  |  |
| Current treatment | | | | | | | | | | | | | | | | | | | | | |  |  |
|  | CT | | 58.2 ± 22.1 | | | 66.3 ± 25.0 | | | | 70.0 ± 38.0 | | | 70.5 ± 30.1 | | | 75.4 ±27.2 | | | 80.0 ± 30.2 | | |  |  |
|  | S | | 62.8 ± 23.8 | | | 71.4 ± 23.1 | | | | 70.8 ± 36.2 | | | 66.3 ± 35.2 | | | 86.1 ± 16.8 | | | 82.6 ± 29.7 | | |  |  |
|  | HT | | 62.5 ± 23.3 | | | 70.6 ± 24.1 | | | | 81.5 ± 30.6 | | | 76.4 ± 26.6 | | | 82.8 ± 25.1 | | | 82.5 ± 27.5 | | |  |  |
|  | RT | | 49.1 ± 23.7 | | | 64.4 ± 31.6 | | | | 58.0 ± 39.9 | | | 59.8 ± 33.1 | | | 74.7 ± 27.3 | | | 66.7 ± 38.6 | | |  |  |
|  | | *p*-value | 0.023* | | | 0.330 | | | | 0.002* | | | 0.026* | | | 0.024* | | | 0.077 | | |  |  |
| Comorbid conditions | | | | | | | | | | | | | | | | | | | | | |  |  |
|  | Yes | | 61.8 ± 21.7 | | | 65.3 ± 24.8 | | | | 78.3 ± 34.0 | | | 73.8 ± 29.5 | | | 75.4 ± 26.9 | | | 81.9 ± 30.7 | | |  |  |
|  | No | | 58.6 ± 23.2 | | | 68.7 ± 25.2 | | | | 71.8 ± 36.7 | | | 70.9 ± 29.8 | | | 79.4 ± 26.0 | | | 79.6 ± 29.9 | | |  |  |
|  | | *p*-value |  | | 0.256 | | | | 0.276 | | | 0.140 | | | 0.415 | | | 0.208 | | | 0.509 | | |

PF=Physical Functioning, RF=Role functioning, EF=Emotional functioning, CF =Cognitive functioning, SF=Social functioning, CT= Chemo therapy, S= Surgery, HT= Hormonal therapy, RT=Radiotherapy

*The mean difference is significant at < 0.05 (ANOVA)

Table S3: Mean differences of EORTC QLQ-C30 symptom scale with socio-demographic/socio-economic characteristics of patients with breast cancer at TASH, Addis Ababa, Ethiopia, 2018.

|  | | | | Fatigue | Nausea/Vomiting | Pain | Dyspnea | Insomnia | Appetite loss | Constipation | Diarrhea | Financial Difficulties |
| --- | --- | --- | --- | --- | --- | --- | --- | --- | --- | --- | --- | --- |
| Age(years) | | | | | | | | | | | | |
|  | | 15-24 | | 22.2± 22.2 | 5.6±9.6 | 27.8±25.4 | 22.2±19.2 | 44.4±50.9 | 22.2±38.5 | 22.2±38.5 | 0.00±0.00 | 66.7±33.3 |
|  | | 25-54 | | 41.3±33.3 | 14.5±25.3 | 36.4±32.9 | 17.2±29.4 | 31.9±39.8 | 35.8±40.1 | 24.9±35.9 | 4.3±15.4 | 48.5±44.8 |
|  | | 55-64 | | 43.1±32.9 | 12.6±20.7 | 33.0±33.2 | 15.8±26.0 | 33.9±36.4 | 30.9±40.3 | 22.8±35.2 | 2.3±10.6 | 48.5±43.6 |
|  | | >65 | | 57.4±32.7 | 20.1±29.9 | 45.8±33.4 | 44.4±45.7 | 47.2±46.0 | 59.7±43.9 | 29.2±35.9 | 5.5± 16.0 | 47.2±46.0 |
|  | | | *p*-value | 0.097 | 0.863 | 0.430 | 0.012* | 0.324 | 0.031* | 0.815 | 0.666 | 0.916 |
| Marital status | | | | | | | | | | | | |
|  | | Single | | 40.9±31.6 | 11.0±23.8 | 36.6±33.1 | 15.5±25.4 | 33.9±40.9 | 27.4±38.2 | 29.8±41.5 | 2.9±11.5 | 45.2±47.3 |
|  | | Married | | 43.0±33.8 | 15.2±25.7 | 36.6±32.9 | 18.4±31.4 | 34.0±40.4 | 38.4±40.3 | 26.1±35.9 | 4.6±16.9 | 46.8±43.3 |
|  | | Divorced | | 36.9±31.9 | 13.9±21.9 | 32.1±31.7 | 15.5±23.7 | 23.2±32.4 | 34.5±41.7 | 20.2±30.9 | 2.9±9.6 | 52.9±45.3 |
|  | | Widowed | | 46.5±34.5 | 15.2±26.0 | 39.7±34.2 | 25.5±37.0 | 38.3±42.4 | 39.4±43.2 | 19.4±32.6 | 3.9±12.4 | 54.4±46.3 |
|  | | | *p*-value | 0.456 | 0.581 | 0.670 | 0.544 | 0.334 | 0.268 | 0.477 | 0.961 | 0.523 |
| Level of education | | | | | | | | | | | | |
|  | | Illiterate | | 49.6±32.8 | 17.9±25.9 | 40.6±32.3 | 25.7±36.3 | 39.5±41.0 | 48.9±40.3 | 32.9±40.6 | 3.3±11.1 | 58.3±44.4 |
|  | | Informal education | | 38.5±32.0 | 14.9±21.9 | 33.9±30.2 | 16.7±30.8 | 26.2±37.8 | 38.1±41.3 | 27.4±32.8 | 2.4±8.7 | 42.8±43.4 |
|  | | Primary education | | 43.1±32.6 | 13.4±24.5 | 37.3±32.9 | 15.8±24.0 | 33.3±39.2 | 32.0±39.8 | 25.9±35.9 | 3.5±15.9 | 51.3±43.7 |
|  | | Secondary education | | 41.4±34.1 | 12.9±23.9 | 36.8±34.2 | 18.1±30.8 | 33.3±41.1 | 37.1±41.2 | 18.7±31.7 | 2.9±12.1 | 50.7±45.2 |
|  | | Higher education | | 36.6±33.2 | 13.9±26.8 | 31.6±32.5 | 14.9±28.4 | 28.2±37.6 | 25.5±38.0 | 23.1±35.3 | 7.4±20.8 | 34.5±42.2 |
|  | | | *p*-value | 0.114 | 0.419 | 0.472 | 0.267 | 0.316 | 0.002* | 0.099 | 0.445 | 0.007* |
| AMHI, in ETB | | | |  |  |  |  |  |  |  |  |  |
|  | ≤600 | | | 46.1±34.3 | 17.8±25.3 | 40.3±34.8 | 20.7±31.5 | 32.8±38.6 | 40.6±41.0 | 31.5±39.1 | 2.8±11.0 | 60.7±43.8 |
|  | >600 | | | 40.6±32.8 | 12.9±24.7 | 34.7±31.9 | 17.7±30.3 | 33.3±40.5 | 34.5±40.5 | 21.7±33.6 | 4.6±16.2 | 42.9±43.8 |
|  | | | *p*-value | 0.127 | 0.012* | 0.108 | 0.323 | 0.929 | 0.156 | 0.019* | 0.450 | 0.000* |

*The mean difference is significant at<0.05 (ANOVA)

Table S4: Mean differences of EORTC QLQ-C30 symptom scale with clinical characteristics of patients with breast cancer at TASH, Addis Ababa, Ethiopia, 2018.

|  | | | | Fatigue | | Nausea and vomiting | | | Pain | | | | | Dyspnea | | | Insomnia | | | Appetite loss | | | Constipation | | | Diarrhea | | | Financial Difficulties | | |  |  |
| --- | --- | --- | --- | --- | --- | --- | --- | --- | --- | --- | --- | --- | --- | --- | --- | --- | --- | --- | --- | --- | --- | --- | --- | --- | --- | --- | --- | --- | --- | --- | --- | --- | --- |
| Patient status | | | | | | | | | | | | | | | | | | | | | | | | | | | | | | | |  |  |
|  | New patient | | | 39.3±33.4 | | 13.2±22.6 | | | 41.9±34.2 | | | | | 19.4±31.0 | | | 34.9±41.1 | | | 39.5±44.9 | | | 24.8±34.2 | | | 2.3±11.2 | | | 36.4±41.7 | | |  |  |
|  | Follow up | | | 42.7±33.3 | | 14.6±25.2 | | | 35.8±32.7 | | | | | 18.5±30.7 | | | 32.9±39.7 | | | 36.1±40.2 | | | 24.8±35.9 | | | 4.2±15.1 | | | 50.0±44.7 | | |  |  |
|  | | *P*-value |  | 0.519 | | | | 0.893 | | | 0.256 | | | | | 0.907 | | | 0.755 | | | 0.694 | | | 0.830 | | | 0.353 | | | 0.058 | | |
| Time since diagnosis (months) | | | | | | | | | | | | | | | | | | | | | | | | | | | | | | | |  |  |
|  | <12 | | | 42.2±33.7 | | 19.4±26.9 | | | 38.9±33.9 | | | | | 19.1±30.9 | | | 33.3±39.5 | | | 42.7±40.6 | | | 22.4±33.7 | | | 5.9±17.9 | | | 48.8±44.8 | | |  |  |
|  | 13-60 | | | 41.8±33.8 | | 9.4±21.9 | | | 33.5±31.8 | | | | | 18.2±30.9 | | | 33.1±40.4 | | | 28.6±40.0 | | | 28.3±37.7 | | | 1.9±10.2 | | | 49.6±43.8 | | |  |  |
|  | >60 | | | 45.9±29.9 | | 7.2±18.7 | | | 34.7±31.5 | | | | | 18.0±28.9 | | | 32.4±40.4 | | | 33.3±38.5 | | | 24.3±38.2 | | | 1.8±7.6 | | | 43.2±47.0 | | |  |  |
|  | | *P*-value |  | 0.788 | | | | 0.000* | | | 0.291 | | | | | 0.925 | | | 0.977 | | | 0.001* | | | 0.337 | | | 0.034* | | | 0.737 | | |
| Stage of cancer | | | | | | | | | | | | | | | | | | | | | | | | | | | | | | | |  |  |
|  | Stage1 | | | 41.9±32.1 | | 11.5±24.9 | | | | | | 37.2±25.6 | | 7.7±19.9 | | | 38.5±38.1 | | | 28.2±38.1 | | | 23.1±34.4 | | | 0.00±0.00 | | | 46.1±46.2 | | |  |  |
|  | Stage2 | | | 37.5±32.2 | | 10.9±22.0 | | | | | | 28.5±27.9 | | 15.4±27.6 | | | 29.3±39.0 | | | 30.3±37.8 | | | 23.1±33.0 | | | 3.5±13.7 | | | 45.8±43.9 | | |  |  |
|  | Stage3 | | | 38.6±32.4 | | 13.5±23.9 | | | | | | 35.2±32.2 | | 17.4±30.2 | | | 29.1±37.8 | | | 37.1±40.9 | | | 22.1±35.9 | | | 3.9±15.6 | | | 48.1±44.8 | | |  |  |
|  | Stage4 | | | 57.0±34.2 | | 24.0±30.9 | | | | | | 53.9±36.7 | | 29.4±35.9 | | | 47.2±42.1 | | | 47.2±43.0 | | | 33.3±40.4 | | | 6.7±17.7 | | | 53.6±45.1 | | |  |  |
|  | Unknown | | | 41.2±31.4 | | 9.7±17.6 | | | | | | 29.0±31.9 | | 13.9±28.2 | | | 27.9±40.4 | | | 34.4±42.6 | | | 22.6±31.5 | | | 1.1±5.9 | | | 50.5±45.4 | | |  |  |
|  | | *P*-value |  | 0.000* | | | 0.007* | | | | | | 0.000* | | 0.007* | | | 0.006* | | | 0.086 | | | 0.267 | | | 0.094 | | | 0.792 | | |  |
| Current treatment | | | | | | | | | | | | | | | | | | | | | | | | | | | | | | | |  |  |
|  | CT | | | 45.2±33.2 | | 19.5±28.1 | | | 40.1±33.8 | | | | | 19.9±30.9 | | | 37.7±40.0 | | | 44.0±41.1 | | | 26.2±36.2 | | | 6.3±18.7 | | | 51.4±44.4 | | |  |  |
|  | S | | | 34.2±33.6 | | 8.3±16.3 | | | 33.3±31.8 | | | | | 18.0±31.0 | | | 22.2±40.1 | | | 36.1±46.0 | | | 22.2±30.6 | | | 2.8±13.6 | | | 31.9±41.1 | | |  |  |
|  | HT | | | 38.4±31.8 | | 7.7±19.7 | | | 29.7±29.7 | | | | | 15.6±29.6 | | | 28.1±38.1 | | | 25.9±37.9 | | | 23.0±35.6 | | | 0.9±5.6 | | | 47.2±44.8 | | |  |  |
|  | RT | | | 47.1±39.9 | | 15.5±20.4 | | | 44.8±37.6 | | | | | 24.1±34.4 | | | 33.3±43.6 | | | 32.2±36.2 | | | 25.3±37.4 | | | 3.4±10.3 | | | 48.3±45.9 | | |  |  |
|  | | *P*-value |  | 0.139 | | 0.000* | | | 0.014* | | | | | 0.291 | | | 0.035* | | | 0.000* | | | 0.806 | | | 0.015* | | | 0.228 | | |  |  |
| Comorbid conditions | | | | | | | | | | | | | | | | | | | | | | | | | | | | | | | |  |  |
|  | Yes | | | 43.3±32.8 | | 12.6±23.3 | | | 34.3±31.9 | | | | | 24.0±32.6 | | | 32.9±37.4 | | | 33.3±39.6 | | | 24.8±36.9 | | | 5.8±17.8 | | | 45.7±43.7 | | |  |  |
|  | No | | | 42.1±33.5 | | 14.9±25.4 | | | 37.1±33.2 | | | | | 17.2±30.0 | | | 33.2±40.5 | | | 37.3±40.9 | | | 24.8±35.4 | | | 3.6±13.8 | | | 49.4±44.8 | | |  |  |
|  | | *P*-value |  | | 0.778 | | 0.333 | | | 0.492 | | | | | 0.028* | | | 0.972 | | | 0.388 | | | 0.852 | | | 0.221 | | | 0.503 | | |  |

PF=Physical Functioning, RF=Role functioning, EF=Emotional functioning, CF =Cognitive functioning, SF=Social functioning, CT= Chemo therapy, S= Surgery, HT= Hormonal therapy, RT=Radiotherapy

*The mean difference is significant at < 0.05 (ANOVA)

Table S5: Mean differences of EORTC QLQ-BR23 functional scale with socio-demographic/socio-economic characteristics of patients with breast cancer at TASH, Addis Ababa, Ethiopia, 2018.

|  | | | | | | Body image | | | | | Sexual functioning | | | | | Sexual enjoyment | | | | | Future perspective | | | | | | |  |  |
| --- | --- | --- | --- | --- | --- | --- | --- | --- | --- | --- | --- | --- | --- | --- | --- | --- | --- | --- | --- | --- | --- | --- | --- | --- | --- | --- | --- | --- | --- |
| Age(years) | | | | | | | | | | | | | | | | | | | | | | | | | |  |  |  |  |
|  | 15-24 | | | | | 19.4 ± 17.3 | | | | | 22.2 ± 38.5 | | | | | 66.7 | | | | | 22.2 ± 19.2 | | | | |  |  |  |  |
|  | 25-54 | | | | | 76.1 ± 32.1 | | | | | 20.7 ± 29.4 | | | | | 62.7 ± 31.2 | | | | | 52.3 ± 43.9 | | | | |  |  |  |  |
|  | 55-64 | | | | | 85.5 ± 28.4 | | | | | 7.9 ± 20.4 | | | | | 80.9 ± 26.2 | | | | | 57.3 ± 40.7 | | | | |  |  |  |  |
|  | >65 | | | | | 79.9 ± 33.6 | | | | | 1.4 ± 6.8 | | | | | 33.3 | | | | | 47.2 ± 40.4 | | | | |  |  |  |  |
|  | | | | *p*-value |  | | | 0.003* | | | | | 0.000* | | | | | 0.357 | | | | | 0.467 | | | | |  |  |
| Marital status | | | | | | | | | | | | | | | | | | | | | | | | | |  |  |  |  |
|  | Single | | | | | 72.9 ± 35.4 | | | | | 11.3± 21.6 | | | | | 54.5 ± 22.5 | | | | | 48.8 ± 46.7 | | | | |  |  |  |  |
|  | Married | | | | | 76.2 ± 33.0 | | | | | 27.2 ± 31.5 | | | | | 63.4 ± 31.8 | | | | | 53.6 ± 43.2 | | | | |  |  |  |  |
|  | Divorced | | | | | 82.6 ± 25.6 | | | | | 3.3 ± 13.3 | | | | | 83.0 ± 19.2 | | | | | 56.5 ± 42.1 | | | | |  |  |  |  |
|  | Widowed | | | | | 79.9 ± 30.5 | | | | | 1.1 ± 6.8 | | | | | 100 | | | | | 47.8 ± 40.9 | | | | |  |  |  |  |
|  | | | | *p*-value |  | | | | 0.364 | | | | | 0.000* | | | | | 0.269 | | | | | 0.623 | | | | |  |
| Level of education | | | | | | | | | | | | | | | | | | | | | | | | | |  |  |  |  |
|  | | Illiterate | | | | 75.2 ± 34.3 | | | | | 11.4 ± 24.3 | | | | | 66.7 ± 26.3 | | | | | 51.8 ± 44.0 | | | | |  |  |  |  |
|  | | Informal | | | | 86.9 ± 27.6 | | | | | 15.5 ± 29.7 | | | | | 57.1 ± 25.2 | | | | | 64.3 ± 36.2 | | | | |  |  |  |  |
|  | | Primary | | | | 78.6 ± 29.6 | | | | | 13.8 ± 26.3 | | | | | 57.9 ± 31.1 | | | | | 48.7 ± 41.9 | | | | |  |  |  |  |
|  | | Secondary | | | | 73.7 ± 33.9 | | | | | 21.0 ± 29.2 | | | | | 64.5 ± 31.4 | | | | | 52.0 ± 44.2 | | | | |  |  |  |  |
|  | | Higher | | | | 80.0 ± 29.9 | | | | | 24.3± 29.8 | | | | | 64.9 ± 34.2 | | | | | 52.3 ± 44.0 | | | | |  |  |  |  |
|  | |  | | *p*-value |  | | | | | 0.271 | | | | | 0.001* | | | | | 0.879 | | | | | 0.602 | | | | |
| AMHI, in ETB | | | | | |  | | | | |  | | | | |  | | | | |  | | | | |  |  |  |  |
|  | | | ≤600 | | | 78.5 ± 31.6 | | | | | 10.1 ± 22.6 | | | | | 63.9 ± 29.3 | | | | | 35.0 ± 43.0 | | | | |  |  |  |  |
|  | | | >600 | | | 76.6 ± 32.3 | | | | | 21.4 ± 29.7 | | | | | 63.4 ± 31.5 | | | | | 51.3 ± 43.2 | | | | |  |  |  |  |
|  | | | | *p*-value |  | | 0.564 | | | | | 0.000* | | | | | 0.948 | | | | | 0.414 | | | | |  |  |  |

*The mean difference is significant at < 0.05 (ANOVA)

Table S6: Mean differences in EORTC QLQ-BR23 functional scale with clinical characteristics of patients with breast cancer at TASH, Addis Ababa, Ethiopia, 2018.

|  | | | | | Body image | Sexual functioning | Sexual enjoyment | Future perspective |
| --- | --- | --- | --- | --- | --- | --- | --- | --- |
| Patient status | | | | | | | | |
|  | New patient | | | | 65.5 ± 34.9 | 19.8 ± 31.1 | 68.9 ± 34.4 | 30.2 ± 38.4 |
|  | Follow up | | | | 78.6 ± 31.5 | 17.5 ± 27.7 | 62.8 ± 30.6 | 55.1 ± 42.9 |
|  | | | *p*-value |  | 0.011* | 0.746 | 0.477 | 0.000* |
| Time since diagnosis (months) | | | | | | | | |
|  | <12 | | | | 78.2 ± 30.2 | 16.7 ± 28.8 | 62.8 ± 30.1 | 50.5 ± 43.0 |
|  | 13-60 | | | | 77.1 ± 33.6 | 20.2 ± 27.8 | 63.7 ± 32.9 | 55.6 ± 43.1 |
|  | >61 | | | | 72.0 ± 36.6 | 13.9 ± 24.7 | 66.7 ± 27.2 | 50.4 ± 44.2 |
|  | | | *p*-value |  | 0.567 | 0.223 | 0.933 | 0.516 |
| Stage of cancer | | | | | | | | |
|  | | Stage 1 | | | 75.0 ±30.2 | 15.4 ± 25.9 | 50.0 ± 19.2 | 35.9 ± 41.8 |
|  | | Stage 2 | | | 77.9 ± 31.6 | 17.5 ± 28.6 | 65.8± 29.7 | 55.9 ± 43.4 |
|  | | Stage 3 | | | 76.3 ± 32.5 | 20.5 ± 29.0 | 58.9 ± 32.7 | 53.0 ± 42.9 |
|  | | Stage 4 | | | 81.1 ± 32.2 | 13.1 ± 25.5 | 71.9 ± 33.8 | 48.0 ± 43.7 |
|  | | Undefined | | | 68.3 ± 32.7 | 19.9 ± 29.0 | 66.7 ± 24.6 | 53.8 ± 41.9 |
|  | | | *p*-value |  | 0.418 | 0.277 | 0.464 | 0.442 |
| Current treatment | | | | | | | | |
|  | CT | | | | 78.5 ± 32.3 | 16.5 ± 27.6 | 66.7 ± 29.7 | 52.9 ± 43.4 |
|  | S | | | | 63.5 ± 32.9 | 27.8 ± 35.3 | 75.7 ± 21.6 | 34.7 ± 42.2 |
|  | HT | | | | 76.7 ± 32.7 | 18.5 ± 27.8 | 56.8 ± 34.2 | 56.8 ± 41.9 |
|  | RT | | | | 81.0 ± 24.9 | 15.5 ± 25.9 | 58.3 ± 29.5 | 42.5 ± 44.4 |
|  | | | *p*-value |  | 0.160 | 0.369 | 0.199 | 0.070 |
| Comorbid conditions | | | | | | | | |
|  | Yes | | | | 75.2 ± 32.9 | 8.3 ± 18.6 | 62.2 ± 24.8 | 49.2 ± 42.4 |
|  | No | | | | 77.7 ± 31.9 | 20.3 ± 29.7 | 63.7 ± 31.8 | 53.3 ± 43.3 |
|  | | | *p*-value |  | 0.513 | 0.001* | 0.864 | 0.432 |

CT= Chemo therapy, S= Surgery, HT= Hormonal therapy, RT=Radiotherapy

*The mean difference is significant at<0.05 (ANOVA)

Table S7: Mean differences in EORTC QLQ-BR23 symptom scale with clinical characteristics of patients with breast cancer at TASH, Addis Ababa, Ethiopia, 2018.

|  | | | | | Systemic therapy side effects | | | | Breast symptoms | | | | Arm symptoms | | | | Upset by hair loss | | | |  |  |  |
| --- | --- | --- | --- | --- | --- | --- | --- | --- | --- | --- | --- | --- | --- | --- | --- | --- | --- | --- | --- | --- | --- | --- | --- |
| Patient status | | | | | | | | | | | | | | | | | | | | |  |  |  |
|  | New patient | | | | 25.1 ± 18.8 | | | | 27.9 ± 26.8 | | | | 26.1 ± 23.6 | | | | 22.2 ± 38.5 | | | |  |  |  |
|  | Follow up | | | | 35.2 ± 22.8 | | | | 17.3 ± 21.9 | | | | 24.8 ± 25.2 | | | | 26.9 ± 40.4 | | | |  |  |  |
|  | | | *p*-value |  | | | 0.006* | | | | 0.002* | | | | 0.475 | | | | 0.854 | | | |  |
| Time since diagnosis (in month) | | | | | | | | | | | | | | | | | | | | |  |  |  |
|  | <12 | | | | 37.3 ± 22.6 | | | | 20.9 ± 23.7 | | | | 24.9 ± 25.2 | | | | 23.9 ± 38.0 | | | |  |  |  |
|  | 13-60 | | | | 30.7 ± 22.6 | | | | 16.5 ± 21.9 | | | | 25.3 ± 25.5 | | | | 34.9 ± 45.9 | | | |  |  |  |
|  | >61 | | | | 30.2 ± 20.1 | | | | 11.3 ± 17.1 | | | | 23.1 ± 22.7 | | | | 30.9 ± 42.3 | | | |  |  |  |
|  | | *p*-value | |  | | | | 0.012* | | | | 0.006* | | | | 0.963 | | | | 0.419 | | | |
| Stage of cancer | | | | | | | | | | | | | | | | | | | | |  |  |  |
|  | Stage 1 | | | | 29.3 ± 17.6 | | | | 17.9 ± 16.3 | | | | 29.0 ± 20.5 | | | | 11.1 ± 19.2 | | | |  |  |  |
|  | Stage 2 | | | | 30.6 ± 20.9 | | | | 14.6 ± 17.2 | | | | 22.3 ± 22.6 | | | | 25.0 ± 40.3 | | | |  |  |  |
|  | Stage 3 | | | | 33.9 ± 22.2 | | | | 17.6 ± 23.1 | | | | 23.7 ± 24.4 | | | | 33.8 ± 42.7 | | | |  |  |  |
|  | Stage 4 | | | | 41.2 ± 24.0 | | | | 27.2 ± 28.3 | | | | 32.5 ± 29.4 | | | | 22.7 ± 38.1 | | | |  |  |  |
|  | Unknown | | | | 32.9 ± 25.7 | | | | 14.8 ± 22.0 | | | | 19.3 ± 23.9 | | | | 13.3 ± 32.2 | | | |  |  |  |
|  | | | *p*-value |  | | | | 0.016* | | | | 0.013* | | | | 0.044* | | | | 0.440 | | | |
| Current treatment | | | | | | | | | | | | | | | | | | | | |  |  |  |
|  | CT | | | | 42.6 ± 21.4 | | | | 19.6 ± 23.3 | | | | 25.7 ± 25.2 | | | | 27.0 ± 40.3 | | | |  |  |  |
|  | S | | | | 20.8 ± 20.4 | | | | 26.0 ± 27.9 | | | | 27.3 ± 25.8 | | | | 0.0 | | | |  |  |  |
|  | HT | | | | 23.7 ± 19.2 | | | | 14.3 ± 20.2 | | | | 22.9 ± 24.7 | | | | 66.7 ± 57.7 | | | |  |  |  |
|  | RT | | | | 33.0 ± 22.3 | | | | 22.9 ± 22.8 | | | | 26.8 ± 25.6 | | | | 14.8 ± 29.4 | | | |  |  |  |
|  | | | *p*-value |  | | 0.000* | | | | 0.009* | | | | 0.552 | | | | 0.304 | | | |  |  |
| Comorbid conditions | | | | | | | | | | | | | | | | | | | | |  |  |  |
|  | Yes | | | | 33.8 ± 22.9 | | | | 19.6 ± 25.0 | | | | 28.7 ± 28.2 | | | | 22.5 ± 39.5 | | | |  |  |  |
|  | No | | | | 34.2 ± 22.5 | | | | 18.1 ± 22.1 | | | | 23.9 ± 24.1 | | | | 28.1 ± 40.5 | | | |  |  |  |
|  | | | *p*-value |  | | 0.897 | | | | 0.951 | | | | 0.274 | | | | 0.353 | | | |  |  |

CT= Chemo therapy, S= Surgery, HT= Hormonal therapy, RT=Radiotherapy

*The mean difference is significant at <0.05 (ANOVA)

**Table S8: Mean differences of EORTC QLQ-BR23 symptom scale with socio-demographic characteristics/socio-economic of patients with breast cancer at TASH, Addis Ababa, Ethiopia, 2018**

|  | | | | Systemic therapy side effects | Breast symptoms | Arm symptoms | Upset by hair loss |
| --- | --- | --- | --- | --- | --- | --- | --- |
| Age (in years) | | | | | | | |
|  | 15-24 | | | 42.8 ± 29.7 | 22.2 ± 9.6 | 22.2 ± 11.1 | 55.6 ± 50.9 |
|  | 25-54 | | | 33.3 ± 22.2 | 18.3 ± 22.8 | 25.0 ± 24.6 | 27.8 ± 40.6 |
|  | 55-64 | | | 33.8 ± 23.4 | 20.0 ± 25.7 | 24.7 ± 26.7 | 10.7 ± 28.8 |
|  | >65 | | | 44.2 ± 29.1 | 14.9 ± 14.5 | 24.5±29.5 | 48.5 ± 45.6 |
|  | | *p*-value |  | 0.128 | 0.761 | 0.947 | 0.016* |
| Marital status | | | | | | | |
|  | Single | | | 34.7 ± 23.2 | 20.8 ± 25.5 | 25.4 ± 23.6 | 33.3 ± 45.2 |
|  | Married | | | 34.9 ± 22.5 | 18.1 ± 23.2 | 25.0 ± 24.5 | 27.1 ± 40.9 |
|  | Divorced | | | 28.1 ± 22.6 | 16.4 ± 18.4 | 21.4 ± 24.2 | 20.0 ± 34.9 |
|  | Widowed | | | 36.1 ± 21.9 | 19.0 ± 21.9 | 27.4 ± 29.2 | 25.0 ± 36.9 |
|  | | *p*-value |  | 0.196 | 0.902 | 0.611 | 0.840 |
| Level of education | | | | | | | |
|  | Illiterate | | | 37.0 ± 24.9 | 21.7 ± 25.7 | 27.5 ± 27.8 | 39.6 ± 44.3 |
|  | Informal education | | | 30.1 ± 18.4 | 17.5 ± 22.0 | 27.4 ± 30.9 | 18.2 ± 34.5 |
|  | Primary education | | | 37.8 ± 21.2 | 20.6 ± 24.4 | 28.5 ± 26.0 | 21.9 ± 37.4 |
|  | Secondary education | | | 33.4 ± 22.9 | 16.3 ± 19.1 | 22.4 ± 23.2 | 29.2 ± 41.2 |
|  | Higher education | | | 29.9 ± 21.5 | 16.2 ± 22.7 | 21.7 ± 20.9 | 20.0 ± 37.9 |
|  | | *p*-value |  | 0.1183 | 0.249 | 0.484 | 0.122 |
| AMHI (in ETB) | | | | | | | |
|  | ≤600 | | | 37.6 ± 23.1 | 18.9 ± 21.4 | 29.0 ± 28.1 | 27.2 ± 40.8 |
|  | >600 | | | 32.4 ± 22.2 | 18.2 ± 23.3 | 22.9 ± 23.3 | 26.8 ± 40.1 |
|  | | *p*-value |  | 0.031* | 0.220 | 0.103 | 0.899 |

*The mean difference is significant at< 0.05 (ANOVA)
